# Surgery for recurrent stress urinary incontinence: the views of surgeons and women

**DOI:** 10.1007/s00192-017-3376-6

**Published:** 2017-06-02

**Authors:** Douglas G. Tincello, Natalie Armstrong, Paul Hilton, Brian Buckley, Christopher Mayne

**Affiliations:** 1 Department of Health Sciences, University of Leicester, Centre for Medicine, University Road, Leicester, LE1 7RH UK; 2 University Hospitals of Leicester NHS Trust, Leicester, UK; 3 Newcastle Upon Tyne Hospitals NHS Foundation Trust, Newcastle, UK; 4 Department of General Practice, School of Medicine, National University of Ireland, Galway, Ireland

**Keywords:** Surgery, Stress urinary incontinence, Treatment, Preference, Qualitative, Randomization

## Abstract

**Introduction and hypothesis:**

The objectives were to explore the views of women with recurrent stress incontinence (SUI) with regard to treatment preferences and the acceptability of randomisation to a future trial, and to survey the views of UK specialists on treatment preferences and equipoise regarding different treatment alternatives.

**Methods:**

An online survey of the British Society of Urogynaecology (BSUG) and British Society of Urological Surgeons (BAUS) was carried out. Qualitative semi-structured interviews with a purposive sample of surgeons and women suffering from recurrent SUI from three UK centres.

**Results:**

Two hundred fifty-six survey replies were received (176 gynaecology; 80 urology). Comparing the treatments offered, urogynaecologists were more likely to offer pelvic floor exercises (*p* < 0.05), and repeat midurethral tape (MUT) (*p* < 0.001). From the Surgical Equipoise Scale (SES) responses, “no preference” was rarely the commonest response. Marked differences for several options existed; midurethral tape dominated responses whenever it appeared. Twenty-one clinicians were interviewed. Treatment preferences were complex, influenced by a range of factors (reason for failure, patient comorbidity, investigations, personal experience, training). A future trial was regarded as important. Eleven women were interviewed. Most had considered more than one option, but felt that decision-making was more a process of elimination rather than a positive process. Randomisation to a study was regarded as unacceptable by most.

**Conclusions:**

No consensus exists among surgeons about preferred treatment options for recurrent SUI, and personal experience and training dominate decision-making. For patients, choices were usually based on an elimination of options, including that of a repeat failed procedure. This contrasts with surgeons, who mostly preferred a repeat MUT above other options. Any future comparative study will be challenging.

## Introduction

Urinary incontinence (UI) in women is a major issue for the NHS and for society. The prevalence and cost of treatment pose a significant healthcare burden with an ageing population. The Leicestershire MRC Incontinence Study reported that over a third of community-dwelling women aged 40 and over had significant urinary symptoms, with 12% experiencing UI weekly [1]. Stress urinary incontinence (SUI) is a common cause of urinary incontinence and surgery is a highly effective option. The placement of a midurethral tape (MUT; retropubic, transobturator route, or single-incision) to support the urethra is the procedure of choice for primary surgery among most clinicians.

An MUT is a minimally invasive medical device that can be inserted under local or general anaesthesia, as day case surgery. Incontinence cure rates are 60–90%, with a small risk of bladder injury (5%), voiding difficulty and a low incidence of long-term tape erosion or extrusion [2]. Systematic reviews demonstrate retropubic and transobturator tapes to be equally effective in the short term [3–6]. Hospital Episode statistics for England for 2014–2015 indicate that continence surgery is a major driver of caseload and resource cost, with 10,511 continence procedures being performed (over 9,500 MUTs; Hospital Episode Statistics for England, 2014–2015) [7].

Despite the effectiveness of MUT, up to 4 in 10 women suffer from persistent incontinence after surgery, or develop a recurrent problem at some point after their primary operation [2]. There is currently no evidence from randomised controlled trials (RCTs)—and very little from any other type of study—to guide clinicians in how to manage recurrent/persistent incontinence, and no guidance on which is the “best” secondary surgical procedure surgery. Recent systematic reviews presented limited evidence [8–10]. No randomised controlled trials (RCT) solely recruited recurrent cases. Subgroup analysis of RCT data was inconclusive for comparisons between retropubic and transobturator MUT, or between MUT and colposuspension. Data from non-randomised studies suggest cure rates of 73–79% and that retropubic MUT may be more effective than transoburator [9, 11].

This lack of evidence is a real problem for gynaecologists and urologists, who are consulted by women suffering from recurrent or persistent SUI, and who must currently base their treatment choice on clinical experience and personal preference alone. The James Lind Alliance Priority Setting Partnership for Continence identified the management of failed primary surgery as one of the ten research priorities in 2010 [12].

Thus, there remains a pressing need for high-quality research addressing this question, but the design of any RCT in this area is likely to be complex. This paper presents a mixed methods study of the views and preferences of both patients and clinicians with regard to the management of failed primary surgery. Our aim was to identify the issues of greatest relevance for both groups to inform the planning and design of future studies comparing options for women requiring further treatment for recurrent or persistent SUI after primary continence surgery.

## Materials and methods

The study was designed to investigate both patients’ and clinicians’ views and preferences with regard to the acceptability, safety and appropriateness of different surgery comparisons so that any future clinical trials comparing secondary surgeries are feasible and effective in terms of recruitment of participants and willingness of clinicians to allow randomisation.

### Clinician surveys

The aim of the clinician surveys was to assess which surgeries may be most appropriate for comparison in any future trial in terms of clinical equipoise and willingness to allow randomisation.

Members of the British Society of Urogynaecology (BSUG) and the section of Female, Neurological and Urodynamic Urology of the British Association of Urological Surgeons (BAUS) were sent invitations to participate in an online survey to gather information about which of the several treatment options for recurrent/persistent incontinence are considered appropriate and preferable by UK clinicians. Potential options included for secondary surgery were: repeat MUT insertion (same route as for primary surgery); repeat MUT insertion (different route); abdominal colposuspension; fascial sling surgery; urethral bulking agents; and artificial urinary sphincter insertion. The survey collected data about current practice preferences regarding what options each respondent currently offered. Depending on which options were offered, respondents were then presented with different comparisons in the form of the Surgical Equipoise Scale (SES) [13]. In the SES, a choice of two options is presented, and the respondent is asked to indicate whether one is preferred, by marking on a ten-point scale, with each option being “strongly preferred” at either end of the scale, and the midpoint of the scale representing “no preference” (i.e. equipoise). The scale has been used to assess the preferences of medical staff with regard to the treatment alternatives in cancer surgery [14], and clinician views regarding the role of urodynamic testing before surgery [15]. Results are usually rendered as three number ratios of “prefer option A (to a greater or lesser extent)”: “no preference”: “prefer option B (to a greater or lesser extent)”.

### Clinician interviews

The survey included an invitation for clinicians to be interviewed about their views and preferences in more detail. All those who indicated an interest in being interviewed were sent written information and asked to confirm their willingness to participate. Purposive sampling was used to ensure that clinicians with a range of the views expressed in the SES were included. Telephone interviews using an interview topic guide developed from discussions within the study team were conducted by an experienced researcher (see acknowledgements). Interviews were audio-recorded, transcribed and anonymised before analysis. Analysis was based on the constant comparative method [16] and facilitated by NVivo software (QSR International, Australia). Transcripts were read in detail and open codes were initially applied to the data line by line. These open codes were then incrementally grouped into organising categories or themes. These categories were modified and checked constantly as further open codes were incorporated as analysis proceeded. The categories and their specifications (the coding scheme) were then programmed into the software. The coding scheme was used to process the data set systematically by assigning each section of text to a category, according to the category specifications.

### Patient interviews

Eligible women were over 18 years old with a diagnosis of recurrent/persistent stress incontinence who were due to have repeat surgery, or had recently completed repeat surgery. Exclusion criteria included previous continence surgery in conjunction with prolapse surgery, an inability to provide written informed consent, and an insufficient understanding of English to participate in the interviews.

Clinicians from six study sites provided written information about the study to all eligible women on their clinic lists. Those willing to participate made contact with the study team directly. Although we had planned to sample purposively from respondents to ensure a spread of women with immediate failed primary surgery and later recurrence, and women who had different primary and secondary procedures, the lower than expected recruitment meant that this was not feasible.

Written informed consent was obtained from each woman and telephone interviews were conducted by the same researcher who interviewed the clinicians (see acknowledgements). The interviews were audio-recorded, transcribed and anonymised before analysis. Interviews were informed by a topic guide developed with input from our patient representative, and two pilot interviews were completed.

The interviews focused on women’s personal experiences of failed primary surgery and decisions about secondary procedures, in addition to their views about any future RCT in this area. To facilitate discussion of the latter, the interviewer described the alternative surgical and non-surgical options and explored women’s views about the safety and acceptability of each, in terms of likely cure rate, and taking into account complications, length of hospital stay and likely recovery time. Of particular interest was exploring which alternative comparisons (e.g. a day case tape operation compared with an inpatient operation with an abdominal incision) were regarded as the most acceptable, and which were the most likely comparisons to encourage (or discourage) patients to take part in a research study comparing them. Data analysis was based on the constant comparative method [16], and facilitated by the use of NVivo software.

### Statistical analysis

Survey data are presented as number (%) of respondents for each question and response. Comparisons of responses by clinical specialty were done using Chi-squared with Yates’ correction where appropriate. Significance was set at the 5% level.

## Results

### Clinician survey

Two hundred and fifty-six survey replies were received, a response rate of 38% overall. Forty respondents were subspecialist urogynaecologists, 136 were gynaecologists with a “special interest” in urogynaecology; 47 were subspecialist urologists; and 33 urologists with a special interest in female urology. Urogynaecology subspecialists complete a 3-year formal training programme, and special interest clinicians complete a shorter, formal training module, which covers primary procedures, but not apical compartment surgery. Urology subspecialty and special interest is less formal. A subspecialist is defined as someone with more than 50% of clinical work in female urology; special interest urologists do less than this.

**Fig. 1:**
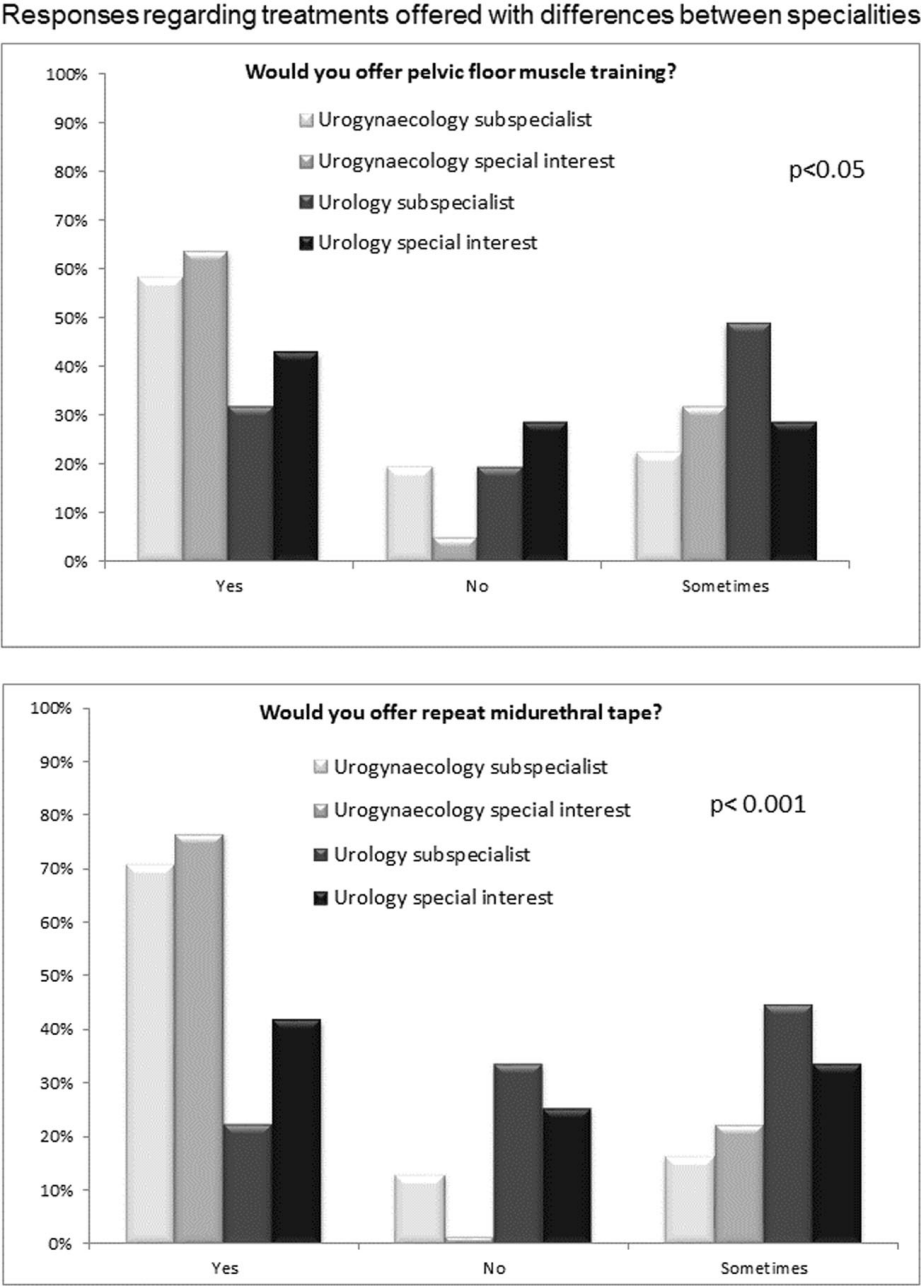
*Bars* indicate percentage response. For actual numbers see Table 2

**Fig. 2:**
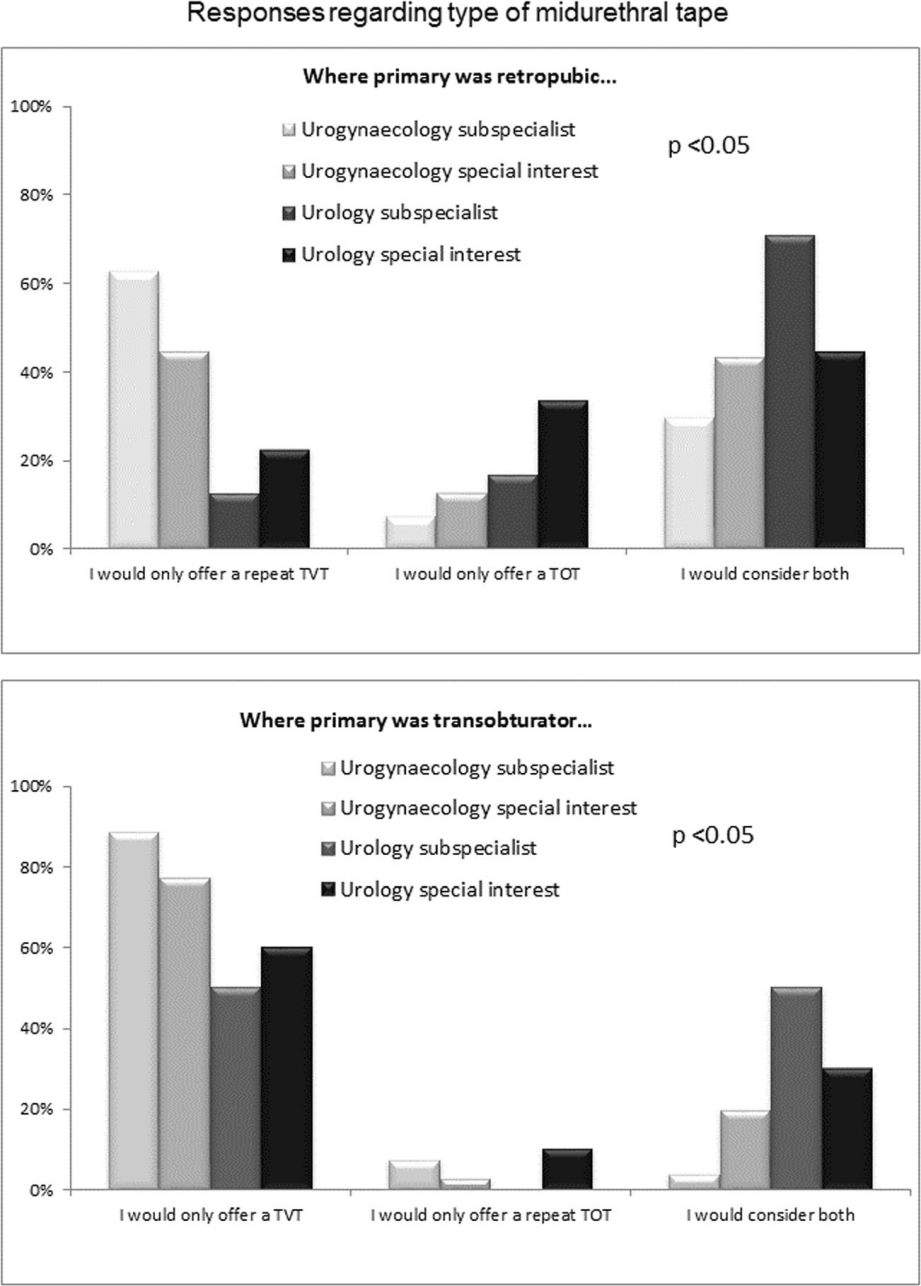
*Bars* indicate percentage response. For actual numbers see Table 3.* TVT* tension-free vaginal tape,* TOT* transobturator tape

Most of the respondents performed repeat continence surgery themselves, but there were differences by specialty, with a greater proportion of subspecialists operating themselves (Table 1). When comparing treatments offered, there were some differences between specialty groups (Fig. 1; Table 2). Single-incision tapes were not offered by many respondents (20 [7.8%] “yes” and 18 [7.0%] “sometimes”). Urogynaecologists were more likely to offer pelvic floor muscle exercises than urologists (*p* < 0.05), and also much more likely to offer a repeat midurethral tape (MUT; *p* < 0.001). Among those offering repeat MUT, there was a clear preference for retropubic tape as second surgery in all cases, with more urologists being willing to consider a transobturator tape in either scenario (*p* < 0.05; Fig. 2; Table 3).

**Table 1:** Treatment patterns among respondents

	Urogynaecology subspecialist	Urogynaecology special interest	Urology subspecialist	Urology special interest	p*
Replies	*n* = 40	*n* = 136	*n* = 47	*n* = 33	
Operate myself	36 (90)	87 (64.0)	43 (91.5)	14 (42.4)	
Refer to another colleague locally	2 (5)	14 (10.3)	1 (2.1)	10 (30.3)	
Refer to a colleague in a secondary level centre	0	3 (2.2)	1 (2.1)	1 (3.0)	<0.001
Refer to a colleague in a tertiary level centre	0	20 (14.7)	1 (2.1)	4 (12.1)	
Other response^a^	2 (5)	12 (8.8)	1 (2.1)	4 (12.1)	

Data are number (%)

*Chi-squared with Yates’ correction for entire table

^a^Examples of text responses included: “…depending on clinical scenario I operate myself or refer to colleague locally…”; “depends on what is decided at multidisciplinary meeting. If pretty much continent, and we are considering bulking agents, I do it. If we think she would benefit from repeat MUT or a fascial sling or other complications, we refer to the tertiary unit”; “Discuss at MDT and operate with colleague”; “I operate jointly with a urogynaecologist on a regular basis and also often with another urologist, both within our own urodynamic MDT structure.”

**Table 2:** Summary of treatments offered by specialty

“Would you offer…for recurrent or persistent incontinence?”		Urogynaecology subspecialist	Urogynaecology special interest	Urology subspecialist	Urology special interest	p*
Pelvic floor muscle exercise	Yes Sometimes No Total	21 (58.3) 8 (22.3) 7 (19.4) 36	52 (63.4) 26 (31.7) 4 (4.9) 82	13 (31.7) 20 (48.8) 8 (19.5) 41	6 (42.8) 4 (28.6) 4 (28.6) 14	<0.05
Bladder neck injection	Yes Sometimes No Total	16 (48.5) 14 (42.4) 3 (9.1) 33	31 (41.3) 29 (38.7) 15 (20.0) 75	15 (39.5) 19 (50.0) 4 (10.5) 38	4 (30.8) 5 (38.4) 4 (30.8) 13	ns
Single-incision tape	Yes Sometimes No Total	4 (12.9) 4 (12.9) 23 (74.2) 31	11 (15.3) 8 (11.1) 53 (73.6) 72	2 (5.6) 4 (11.2) 30 (83.2) 36	3 (25.0) 2 (16.7) 7 (58.3) 12	ns
Burch colposuspension	Yes Sometimes No Total	15 (48.4) 11 (35.5) 5(16.1) 31	25 (34.7) 33 (45.8) 14 (19.5) 72	18 (50.0) 16 (44.4) 2 (5.6) 36	8 (66.7) 3 (25.0) 1 (8.3) 12	ns
Repeat midurethral tape (any type)	Yes Sometimes No Total	22 (71.0) 5 (16.1) 4 (12.9) 31	55 (76.4) 16 (22.2) 1 9 (1.4) 72	8 (22.2) 16 (44.5) 12 (33.3) 36	5 (35.7) 4 (28.6) 5 (35.7) 14	<0.001

Data are number of respondents (%); the number of individual replies varied between questions. See Fig. 1 for proportion of responses with speciality differences

*ns* not significant

*Chi-squared with Yates’ correction

**Table 3:** Questions relating only to repeat midurethral tape option

	Urogynaecology subspecialist	Urogynaecology special interest	Urology subspecialist	Urology special interest	p*
	*n* = 27	*n* = 72	*n* = 24	*n* = 10	
Where the primary tape was retropubic…					
I would only offer repeat retropubic	17 (63.0)	32 (44.4)	3 (12.5)	2 (20.0)	
I would only offer transobturator	2 (7.4)	9 (12.5)	4 (16.7)	3 (30.0)	<0.05
I would offer both	8 (29.6)	31 (43.1)	17 (70.8)	4 (40.0)	
Where the primary tape was transobturator…					
I would only offer a retropubic	24 (88.9)	55 (76.4)	12 (100)	6 (60.0)	
I would only offer repeat transobturator	2 (7.4)	2 (2.8)	0	1 (10.0)	<0.05
I would offer both	1 (3.7)	14 (19.4)	12	3 (30.0)	

Data are number of respondents (%); the number of individual replies varied between questions. See Fig. 2 for proportion responses of responses with specialty differences

*ns* not significant

*Chi-squared with Yates’ correction

One hundred and fifty-two respondents completed the Surgical Equipoise Scales (SES) [13] (28 subspecialist urogynaecologists, 76 special interest gynaecologists, 36 subspecialist urologists and 12 special interest urologists. The design of the survey meant that not all respondents had access to every question. For all possible head to head comparisons, it was noticeable that the “no preference” response (i.e. the comparison was considered to be in equipoise) was almost never the most common one. Only the special interest urologists, on two comparisons, were more likely to be in equipoise, and these numbers were small (colposuspension vs fascial sling, and repeat MUT vs fascial sling). Second, there were marked differences between specialties for several options (Table 4). For bladder neck injection vs pelvic floor exercises, the special interest urogynaecologists were more in favour of exercises. For both major procedure options vs exercises (colposuspension or fascial sling), and fascial sling vs bladder neck injection, the urologists were more likely to favour the major surgery. In relation to major surgery, urologists were more likely to be in favour of fascial sling surgery than colposuspension.

**Table 4:** Responses to different options presented in the Surgical Equipoise Scale

“A” vs “B”	Urogynaecology subspecialist (*n* = 28)	Urogynaecology special interest (*n* = 76)	Urology subspecialist (*n* = 36)	Urology special interest (*n* = 12)	p*
BNI vs PFE	10: 7: 11	22: 6: 48	18: 7: 7	4: 2: 3	<0.02
Fascial sling vs PFE	14: 4: 10	23: 12: 41	25: 4: 3	9: 0: 0	<0.001
PFE vs SIS	15: 3: 9	34: 10: 27	18: 7: 5	2: 3: 4	ns
Colposuspension vs PFE	20: 2: 5	36: 3: 32	21: 5: 4	9: 0: 0	<0.02
Fascial sling vs BNI	18: 2: 8	18: 10: 29	24: 4: 4	6: 1: 2	<0.01
Colposuspension vs BNI	21: 1: 6	26: 10: 21	21: 4: 7	6: 1: 2	ns
BNI vs SIS	13: 10: 5	31: 14: 12	20: 10: 2	4: 3: 1	ns
Colposuspension vs fascial sling	13: 6: 7	40: 11: 7	8: 10: 16	3: 6: 2	<0.01
Repeat MUT vs PFE	21: 4: 2	48: 7: 16	16: 5: 3	9: 0: 0	ns
Repeat MUT vs BNI	19: 5: 3	47: 12: 12	16: 4: 4	6: 1: 2	ns
Repeat MUT vs SIS	23: 2: 2	59: 6: 5	23: 1: 0	7: 2: 0	ns
Repeat MUT vs colposuspension	17: 7: 3	45: 17: 8	10: 7: 7	2: 3: 4	ns
Repeat MUT vs fascial sling	15: 8: 4	52: 15: 3	6: 6: 12	2: 4: 3	<0.001

Responses are presented as number of replies, laid out as: prefer option A: no preference: prefer option B. For all comparisons, “A” is the first option and “B” the second option given in the first column

*BNI* bladder neck injection,* PFE* pelvic floor exercises,* SIS* single-incision tape,* MUT* mid-urethral tape,* ns* not significant

*Chi-squared with Yates’ correction comparing equipoise responses across the four specialty groupings

There was some degree of consensus on a number of issues. It was clear that for every SES presented with repeat mid-urethral tape as an option, that this dominated every time (last five rows of Table 4). It was noticeable that for repeat tape vs colposuspension or fascial sling that urologists were more likely to be in favour of the major surgery and were overall more in equipoise about these two comparisons. Also, when presented with an SES regarding how to deal with the existing (failed) midurethral tape, there was a clear preference (78%) for leaving this in position (remove tape 20: no preference 13: leave tape 116) with no significant difference between specialities (*p* = 0.167). Among the 40 respondents who offered single-incision tapes, most preferred this to a colposuspension (57.5%) and fascial sling (57.5%), with no difference between specialities.

### Clinician interviews

Twenty-one clinicians were interviewed: 2 general urologists, 5 urologists with a subspecialist interest, 7 subspecialist urogynecologists and 7 urogynecologists with a special interest. Discussion of current practice supported the variation observed from the survey responses. Treatment preferences were complex and influenced by a range of factors including: how/why the first procedure had failed; time since the first procedure; patient-related factors such as weight or other co-morbidities; patient preferences for treatment; severity of the incontinence; and the results of the urodynamics and other investigations.

The overall preference for a repeat tape observed in the survey was explained by both those who expressed this preference and those commenting on others’ practice as being primarily due to this being a relatively easy and readily available option with which people were experienced and comfortable:

[I do it] because it’s easy. I mean I’ve done a couple, just sort of snipped out the middle bit of the tape that was there…and put another one in, and so far they’ve done, they’ve done well. (Participant S01)

My suspicion is that, you know, some people, rather than referring them to somebody else who might be able to offer these other options, they just get a repeat tape…and of course a tape is a good operation, you know, it’s got a good benefit risk profile, you know, it’s not very interventional, and it’s got, and it’s got good successes. (Participant S12)

The declining expertise in the more invasive procedures was commonly discussed as an important factor underlying the preference for repeat tapes:

There’s only [a] percentage of people who can do a sling, and it’s quite a small one. And in fact increasingly there will only be a small group that can do Burches [colposuspensions], you know fluently and comfortably, so I mean one of the problems is there are lots of surgeons who have very limited repertoires and so that limits them to what they can do. (Participant S06)

As regards a future RCT, there was general agreement that this is an important research topic, as evidence is needed; only one of the participants would not be willing to be recruited to it. However, views differed on what would be appropriate comparisons in an RCT and how this could best be designed. Different views were expressed about the acceptability of different procedures to include, the acceptability of including minimally invasive or non-surgical options (such as bulking agents or pelvic floor exercises) alongside surgical ones, and the number of trial arms that would be needed. Half of those who were willing to be recruited in principle (10 out of 20) made it clear that this would depend on the treatment options included (but there was no consensus among them on these), and sometimes their lack of experience or confidence with certain procedures would rule them out completely.

### Patient interviews

Eleven women were recruited and interviewed across three study sites. Of these, 5 had recently had their secondary procedure and the other 6 were awaiting theirs; secondary procedures included colposuspension, TVT, TOT, and bulking injections. All but 2 women reported having considered more than one possible procedure and discussing this with both clinical staff and family members. The 2 who did not, said that they were happy to take their clinician’s recommendation without further discussion.

Ultimately though, those who said they looked at more than one possible procedure felt that their options were limited given either their past treatment experiences and/or their personal preferences, such as avoiding a general anaesthetic or a long recovery period:

I didn’t want a lengthy stay, and I didn’t want to go through, all that was at the back of my mind is that if I have that operation again, I might, the wound may not heal like it should…[but]… the main reason why I didn’t want surgery again is because I had a respiratory arrest…that’s why I was glad when the consultant said there’s another thing that he could do. (Participant 03)

Because of these apparent restrictions, most women who reported looking at options talked about their decision-making as being akin to a process of elimination rather than as a more positive process of selection between possible options:

I felt as though everything else had failed…and, you know, I thought well, I’ll try this [colposuspension] I didn’t know what to try next really […] I sort of jumped at it, you know, wholeheartedly, think “ooh, is it going to work? Hopefully it will”. (Participant 05)

Most women were not prepared to consider repeating procedures they had previously undergone, as they regarded these as being failures. The only exception to this was a woman who had undergone a successful colposuspension 20 years ago that had now failed—she was keen to repeat this procedure, as it had been effective for so long with no complications. The distinction between immediate and longer-term failure and the assumption of repeated success are clearly important:

I just wanted something that I knew was going to work. I never considered it [another option], I just wanted something, because I’d had it done before…I knew it was going to last me for the rest of my life. (Participant 08)

Given these preferences about the procedures they did and did not regard as acceptable, it is not surprising that most women did not think randomisation in a future RCT would be acceptable. This was most often framed in terms of them not wanting to be randomised to repeat a procedure that had already failed for them, but factors such as wanting to avoid a general anaesthetic were also mentioned:

Well, like you say, you don’t know what, if it’s just a random treatment, you don’t know what you’re going to end up with, do you? If they’d have said, you know, if they’d said “you’ve got to have another TVT”, I wouldn’t have wanted it. (Participant 01)

Although women were generally not prepared to enter a trial where there was a chance that they could be randomised to a treatment that they had already tried, of the pairings put to them for consideration, randomisation between two larger surgical procedures was the most acceptable. This is probably not surprising given that most had tried less invasive treatments first.

## Discussion

The issue of how to manage recurrent or persistent stress incontinence after the failure of primary surgery is clearly an important and yet contentious topic. Our research has highlighted this, and explored the key issues of importance to clinicians, and most importantly, to women. From a surgeon’s perspective, the survey data clearly demonstrate that the lack of evidence means that current practice is highly variable across the UK, based on personal preference or experience. Most had strong views in favour of one option for almost every comparison we presented, illustrating a lack of equipoise, but in the face of an acknowledged lack of research data to support or inform their position. There were some trends observed, with urologists being more likely to favour major interventions over more minor procedures, whereas gynaecologists had a tendency to prefer less major alternatives such as pelvic floor muscle training or bladder neck injections.

What was most striking was the dominant effect of repeat MUT in every comparison in which it was included, a finding that was confirmed from the interviews as being a consequence of training and experience rather than an actual preference. It appeared that many respondents were unable to offer alternative procedures because they had not received training in procedures such as colposuspension or fascial sling. This is an important finding not only for future research plans, but also as a training and clinical governance issue, bearing in mind the increasing concerns about MUT complications, how they are managed, and the possibility of providing women with alternative choices [17, 18].

From the patients’ perspective, the variability and inconsistency of surgeons’ responses in general is a finding that will generate considerable concern, particularly given that this survey was only sent to those with specific training and a declared interest in pelvic floor dysfunction. Patients hope and expect that doctors know what they are talking about and that treatments offered are the most suitable/effective. However, it is clear from the data that the treatments women may be offered may depend largely upon the discipline and training of the surgeon, and that the choice of treatments offered depends upon the surgeon’s skills, experience and opinion rather than any evidence. This highlights the importance of comprehensive and appropriate training, in addition to the need for research addressing the specific issue of failed continence surgery, to avoid and reduce the variability in patient choice that is currently present and to provide greater consistency of care provision. The patient interviews demonstrated that women often had firm opinions about what options they would consider for future care. These opinions were usually based on eliminating unacceptable options, usually including a repeat of the failed original procedure. Given that most women currently will have received an MUT as primary surgery, this runs contrary to the views of most surgeons, who preferred a repeat MUT above all other options.

From the perspective of designing future research, this study has highlighted several issues. Firstly, and importantly, it is clear that more research is needed to address these uncertainties, a fact that was acknowledged by both the patients and clinicians who participated. Second, there are important issues of research design to consider: for patients, surgical options represent different demands upon them. These range from choosing between day-case versus inpatient surgery, short or longer term recovery from surgery, and different combinations and risks of potential side effects. We have demonstrated that patients feel strongly about avoiding a repeat MUT procedure, especially if they have suffered complications (e.g. erosion into the vagina, leading to the need for excision and hence recurrent incontinence), and many have reservations about the acceptability of randomisation as a concept in this scenario. These issues are vital to consider during the design of any future research, to provide a study design that is acceptable to women, in which they are willing to take part, and that addresses questions of direct relevance to them, and other women in the future. It was unfortunate that we were not able to recruit more women who had experienced failure of their primary surgery for interview. As far as we are able to ascertain, this was because there were fewer eligible women than anticipated across the study sites. However, although we were only able to recruit 11 women for interview, these were a diverse group with a range of experiences and preferences. Therefore, while we cannot be sure that we reached saturation with this number of interviews, this does not undermine the usefulness of the data collected.

It was clear from interviews with clinicians that a questionnaire providing treatment alternatives did not capture some of the complexity of planning appropriate treatment for women after failed surgery and we acknowledge this. Interviewees discussed how patient-specific factors such as obesity, co-morbidity, urethral mobility, and voiding dysfunction would need to be considered, in addition to any preferences expressed by the patient. These factors also need to be addressed in the design of any future study, as a means of comprehensively assessing, and potentially stratifying, allocation to different treatment options. Any planned trial would certainly require multicentre recruitment and thus must be acceptable and meaningful to most of the consultant gynaecologists and urologists in the UK and beyond. Acceptability includes factors such as which alternative procedures should be compared; whether the degree of vaginal scarring and urethral mobility should be taken into account; and what diagnostic tests are required before surgery.

The response rate to the survey was relatively low at 38%, which some may argue reduces the generalisability of the results. We sent two reminders. It is possible that the non-responders were not actively treating women with recurrent incontinence, as the members work across a range of hospital settings within the UK, but we cannot be certain. Despite the low proportion of responses, we believe that the message from the data is clear and do not think that practice would be much different among non-responders.

In conclusion, this study reveals the complexity and difficulty of decision-making for women with recurrent stress incontinence. Women’s preferences are limited by personal preferences, chiefly a wish to avoid a repeat a previously unsuccessful procedure. There is no consensus among surgeons and it is clear for a variety of reasons that most prefer to offer a repeat MUT; thus, there is clear mismatch of expectation, given that MUT is the most common primary continence procedure worldwide. Future comparative studies are needed to provide evidence to support decision-making, but because of perceived differences in treatments available, randomisation to any future trial was unacceptable to most women, except between two major procedures. Furthermore, not all clinicians are able to offer all possible options.
